# Bacterial etiologies, antimicrobial susceptibility pattern and associated factors among patients suspected sterile body site infections at Debre Markos Comprehensive Specialized Hospital, Northwest Ethiopia

**DOI:** 10.3389/fmed.2024.1260841

**Published:** 2024-05-07

**Authors:** Dires Admas, Gebreselassie Demeke, Adane Adugna, Ahmed Esmael

**Affiliations:** Department of Medical Laboratory Science, College of Health Sciences, Debre Markos University, Debre Markos, Ethiopia

**Keywords:** antimicrobial susceptibility pattern, bacterial etiologies, associated factors, sterile body fluids, Ethiopia

## Abstract

**Background:**

Sterile body locations are usually associated with clinical urgency and life-threatening illnesses, and they are typically contaminated with diverse bacterial etiologies. If the bacteria acquire resistance to antimicrobial drugs, the public health crisis will only worsen. In developing countries, drug-resistant bacteria are common because of poor surveillance, diagnostic capacity, and control measures. Early diagnosis, and assessing the drug resistance and factors associated with infection are important to combat the drug resistance and treatment. This study aimed to assess the bacterial etiologies, antimicrobial susceptibility pattern, and possible associated factors among patients suspected of sterile body sites.

**Methods:**

A hospital-based cross-sectional study was conducted from June 2022 to August 2022 at Debre Markos Comprehensive Specialized Hospital in Amhara regional state, Ethiopia. One hundred seven study participants were selected using consecutive convenient sampling techniques. A structured questionnaire was used to collect socio-demographic and clinical data. Gram stain was done for a preliminary report and inoculated into blood agar, MacConkey agar, and chocolate agar and incubated aerobically and micro aerobically at 37°C for 24 h. Antimicrobial susceptibility testing was done by the modified Kirby Bauer’s disk diffusion method. Data were analyzed using bivariate and multivariate logistic regression was used. A *p*-value less than 0.05 is considered as statistically significant.

**Results:**

The overall magnitude of sterile body site infection among study participants was 7.5% (14/187). The majority of the isolates were Gram-negative bacteria with the predominant species *Enterobacter cloacae* accounting for 28.57% (4/14). Among isolates 78.57%(11/14) of them were multidrug-resistant isolates. Being inpatient, co-morbidity, and alcohol consumption were significantly associated with sterile body site infection.

**Conclusion:**

In our study, Gram-negative bacteria were the predominant bacteria that infects sterile body fluid. The prevalence of multi-drug resistance bacteria isolates was significantly high. Therefore, before prescribing an empirical treatment, a medical professional should identify the bacterial etiology of sterile body fluids and the susceptibility of microbes to the drug.

## Introduction

Sterile body fluids (SBF), which include cerebrospinal fluid (CSF), peritoneal fluid, pleural fluid, and synovial fluid, are fluids that have no normal flora of bacteria. Sterile body infection and drug resistance bacteria is life threatening ceases. One of the most urgent public health problems facing the world today is antibiotic resistance. It is typically associated with higher medical costs, longer hospital stays, and more mortality (1, 2). Because of insufficient policy implementation for the prevention and control of drug resistance and laboratory infrastructure inadequacy for obtaining samples and identifying the particular agent, the situation simply gets worse. It was more challenging in those settings since it was necessary to collect samples from invasive locations and confirm the agents for diseases including meningitis, peritonitis, septic arthritis, and pleural empyema (3–8). These circumstances force medical professional to treat patients empirically, which exacerbates the problem of drug resistance already facing public health (1, 2).

Meningitis, peritonitis, septic arthritis, and pleural empyema are only a few of the potentially fatal diseases caused by bacterial infections in sterile body sites (3–8). A worldwide study found that there were 2.5 million cases of meningitis and 236,000 deaths from the disease (9). Also Wunrow et al. (9), bacterial meningitis affects approximately 1.2 million people each year and causes almost 170,000 deaths globally (10). In developing nations, bacterial meningitis continues to pose a serious threat to public health (11). The highest burden of bacterial meningitis occurs in sub-Saharan Africa, known as the meningitis belt (12). Despite the availability of effective antimicrobials, mortality rates in developing nations are higher, ranging from 16 to 32% (13).

Furthermore, spontaneous bacterial peritonitis (SBP) contributes 10 to 30% prevalence and its prevalence even increased among hospitalized patients (25–30%), of which 15% of the infection rate is caused by post-operative procedures (4, 14, 15). This disease is significantly associated with a high level of mortality (90%) (4). Gram-negative flora is the major cause of SBP and mortality rates reach up to 10 to 46% (16). Moreover, bacterial-related pleural infection has increased from 7.6 to 14.9% globally with a 20% mortality rate (15, 17). Also, Septic arthritis is a serious orthopedic issue and the incidence of septic arthritis of native joints has been reported to be around two cases per 100,000 people per year (18, 19). In this regard, the prevalence of bacterial isolates from sterile body fluids in Ethiopia is 11.5–20.2%. Of this, 10.6–40.7% of the isolates were Gram-positive bacteria whereas the rest 9.6–74.6% were Gram-negative bacteria (5–8).

Currently, the emergency of multidrug-resistant (MDR) bacterial infection challenges treatment success and leads to increased mortality (20, 21). MDR bacteria are bacteria that are resistant to three or more classes of antimicrobial drugs. Ethiopia, a developing country in Sub-Saharan Africa, further aggravates the problem with its inadequate laboratory infrastructure, poor antimicrobial surveillance and control measures, and high rates of antimicrobial misuse from drug stores even without a medical prescription (22, 23).

For instance, a study conducted by Frehiwot and colleagues in Ethiopia found a high level of MDR (75.9%) in the bacterial isolates. According to this study 100% and 90% of MDR was from CoNS isolates and *K. pneumoniae* isolates, respectively (6). The main reason for the high prevalence of drug resistance in this setting could be related to a habit of self-medication of antimicrobials from drug stores, poor microbiology facilities, and diagnostic capacity (24). Studies have also shown that surgical procedures, trauma, age, hospitalization, chronic illnesses (HIV/AIDS, hepatitis, cirrhosis, cancer, and diabetes mellitus), as well as other behavioral factors (alcohol misuse and cigarette smoking), are significantly associated to bacterial invasion into the sterile body site (25–27).

Therefore, assessing the prevalence of bacterial etiology, and antimicrobial susceptibility patterns, and addressing potential associated factors of bacterial infection of sterile body sites in settings where poor surveillance and control measures, inadequate laboratory infrastructure, medical professionals treat patients empirically, as well as a lack of published data on this matter, could contribute to better management of bacterial diseases and address factors for the development of drug resistance.

## Materials and methods

### Study setting, design, and population

A hospital-based cross-sectional study was conducted among patients suspected of bacterial sterile body site infections at Debre Markos Comprehensive Specialized Hospital (DMCSH), Northwest Ethiopia, from June 2022 to August 2022 from patients suspected of sterile body fluid infections and had no a history of antimicrobials intake within the last two weeks. Socio-demographic (age, gender, marital status, residence, occupation, income, and educational status), clinical data (history of hospitalization, co-morbidities, cigarette smoking), and other possible associated factors were collected by using a pretested structured questionnaire. DMCSH is found in Debre Markos town and provides specialized health care services through its medical, clinical and diagnostic departments for approximately 255,248 people per year. In the present study, the prevalence of bacterial growth was dependent variable whereas the Socio-demographic data (age, gender, residence, occupation, educational status, and monthly income), Clinical-related data (Patient setting (inpatient, outpatient), history of Hospital admission, history of invasive procedures& surgery, and clinical features), and Co-morbidity (DM, HIV, TB, chronic liver disease, cancer &others) and Behavioral factors (Alcoholism, Smoking cigarettes) were independent variables.

### Sample size and sampling technique

The sample size was determined using a single population proportion formula by considering a prevalence (p) of 14.1% from the Tikur Anbesa Hospital, a 95% confidence interval of Z = (1.96), and a margin of error (d) of 5% (6).

$$n=\frac{\left(z\alpha_{/2}\times P\left(1-P\right)\right)}{d^{2}}.\;\mathrm{So},\;n=\frac{\left(1.96\times 1.96\right)\left(0.141\times 0.859\right)}{0.05\times 0.05}=187$$

Consecutive convenient sampling technique was used to recruit study participants.

### Specimen collection, transportation and bacteriological analysis

One to five mL of sterile body fluid samples (lumbar puncture, paracentesis, thoracentesis, and arthrocentesis) were collected aseptically in sterile glass tubes from each participant by experienced physicians. The sample collection tube was labeled and closed tightly. Then specimens were transported to the hospital laboratory within 10 min and laboratory analyses were started within 30 min of specimen delivery.

#### Inoculation, incubation and isolation of bacterial agent

A drop of the suspension was inoculated into blood agar, chocolate agar, and MacConkey agar plates (Oxoid Ltd., Basingstoke, and Hampshire, United Kingdom). Blood and chocolate agar plates were incubated at 35–37°C in a candle jar with a capnophilic (5–10% CO_2_) environment and MacConkey agar was incubated aerobically for the isolation of Gram-negative aerobic bacteria and examined after 24 h. Mannitol salt agar (Oxoid Ltd., Basingstoke, and Hampshire, United Kingdom) was used to isolate *S. aureus* when beta-hemolysis was observed from the blood agar plate. For mixed colonies, a sub-culture of blood agar and chocolate agar was performed to get pure colonies (28).

#### Identification of bacterial isolates

Isolates were identified by colonial morphology such as hemolytic reaction on blood agar plate, pigment production or color changes surrounding carbohydrate fermenting colonies on MacConkey agar plate. After obtaining pure colonies, further identification was conducted using standard bacteriological techniques including Gram reaction, colony morphology, and biochemical tests. A battery of biochemical test such as catalase test, coagulase test, Optochin sensitivity test, bacitracin sensitivity test, mannitol test, carbohydrate utilization, indole production, mannitol fermentation, citrate utilization, lysine decarboxylation, H2S production, triple sugar iron utilization, and motility testing used to identify Gram positive and Gram negative bacteria responsible for sterile body fluid infections (28).

#### Antimicrobial susceptibility testing

Using a sterile wire loop, up to 3–5 pure colonies from culture plate were packed and emulsified with sterile normal saline in a test tube and compare with turbidity of the 0.5 McFarland standards. The suspension was inoculated and uniformly distributed on Mueller Hinton agar plates using a sterile cotton bud and incubated at 37°C for 15 min. The appropriate disks impregnated with antimicrobial agents were placed on the surface of agar plates (29). For the AST various antibiotic disks such as gentamicin (GN, 10 μg), erythromycin (ERY, 15 μg), clindamycin (CLD, 2 μg) trimethoprim/sulfamethoxazole (SXT, 1.25/23.75 μg), penicillin (P, 10 units), vancomycin (VA, 30 μg), cefoxitin (FOX, 30 μg), tetracycline (TCY, 30 μg), doxycycline (30 μg), and oxacillin (OXA, 1 μg) (Oxoid, LTD, UK) were used for Gram-positive isolates. Ampicillin (AMP, 10 μg), amoxicillin-clavulanic acid (AMC, 30 μg), ceftriaxone (CRO, 30 μg), ceftazidime (CAZ, 30 μg), amikacin (AMK, 30 μg), gentamicin (GN, 10 μg), meropenem (MEM, 10 μg), imipenem (IMP, 10 μg), ciprofloxacin (CIP, 5 μg), trimethoprim/sulfamethoxazole (SXT,1.25/23.75 μg), piperacillin (10 μg), piperacillin /tazobactam (TZB,100/10 μg), tobramycin (TOB 30 μg) (Oxoid, LTD, UK) were used for Gram-negative isolates (30). To assure the quality of laboratory work all aseptic measures were strictly followed. For instance the sterility of prepared culture media was checked by overnight incubating 5% of the batch at 35–37°C and observed for bacterial growth. The prepared culture media, biochemical test, and antimicrobial susceptibility tests were checked by inoculating the American Type Culture Collection (ATCC) reference strains of *Escherichia coli* (ATCC-25922), *Staphylococcus aureus* (ATCC-25923), *Streptococcus pneumoniae* (ATCC 49619), and *Pseudomonas aeruginosa* (ATCC-27853) which were used as positive quality control throughout the study.

### Data analysis and interpretation

Data were entered into Epi-data version 4.6 software and exported to SPSS version 25 for analysis. Antimicrobial susceptibility pattern was analyzed by World Health Organization (WHO) NET 2022 software. Binary logistic regression models were used to predict the relationship between dependent and independent variables. Variables with a *p*-value <0.25 in the bivariate logistic regression analysis were moved to a multivariate logistic regression analysis. Adjusted odds ratios with 95% confidence interval (CI) were used and variables with a *p*-value less than 0.05 in multivariate analysis were considered as statistically significant.

### Ethical considerations

Ethical clearance was obtained from the Ethical Review Committee of the College of Health Sciences, Debre Markos University (Hsc/R/C/Ser/PG/Co/197/11/14), and Amhara Public Health Institute (APHI). Written informed consent and assent were obtained from each participant and parents or legal guardians. Information obtained from this study used only for the purpose of the study.

## Results

### Sociodemographic characteristics

In this study, a total of 187 sterile body fluid samples were collected from patients suspected of bacterial infections. Among enrolled participants, the majority 103/187 (55.1%) of study participants were males, 72/187 (38.5%) were unable to read and write, and 105/187 (56.1%) were rural residents. The median and interquartile range of the age of the study participants were 35 and 26, respectively (Table 1).

**Table 1:** Socio-demographic characteristics of patients suspected of sterile body site infections at DMCSH, from June 2022 to August 2022.

Variables	Categories	Frequency (*n*)	Percentages (%)
Sex	Male	103	55.1%
	Female	84	44.9%
Age	≤20	68	36.4%
	21–40	54	28.9%
	41–60	36	19.2%
	≥61	29	15.5%
Residence	Rural	105	56.1%
	Urban	82	43.9%
Educational status	Unable to read & write	72	38.5%
	Primary school	39	20.9%
	Secondary school	24	12.8%
	Diploma & above	52	27.8%
Occupation	Farmer	76	40.6%
	Merchant	22	11.8%
	Government employee	24	12.8%
	Student	22	11.8%
	Homemaker	22	11.8%
	Daily labor	12	6.4%
	Others	09	4.8%
Marital status	Single	51	27.3%
	Married	121	64.7%
	Divorced	11	5.9%
	Widowed	4	2.1%
Monthly income	≤3,000	118	63.10%
	3,100–5,900	50	26.74%
	≥6,000	19	10.16%
Sub total		187	100%

### Clinical and sample-related characteristics

Out of 187 body fluid samples, CSF was the most frequently encountered sterile body site accounting for 48.7% (91/187) followed by a peritoneal fluid at 27.2%(51/187). The majority of body fluids were clear 78.1% (146/187) while 8.6% (16/187) of body fluids were turbid.

### Prevalence of bacterial isolates

**Figure 1:**
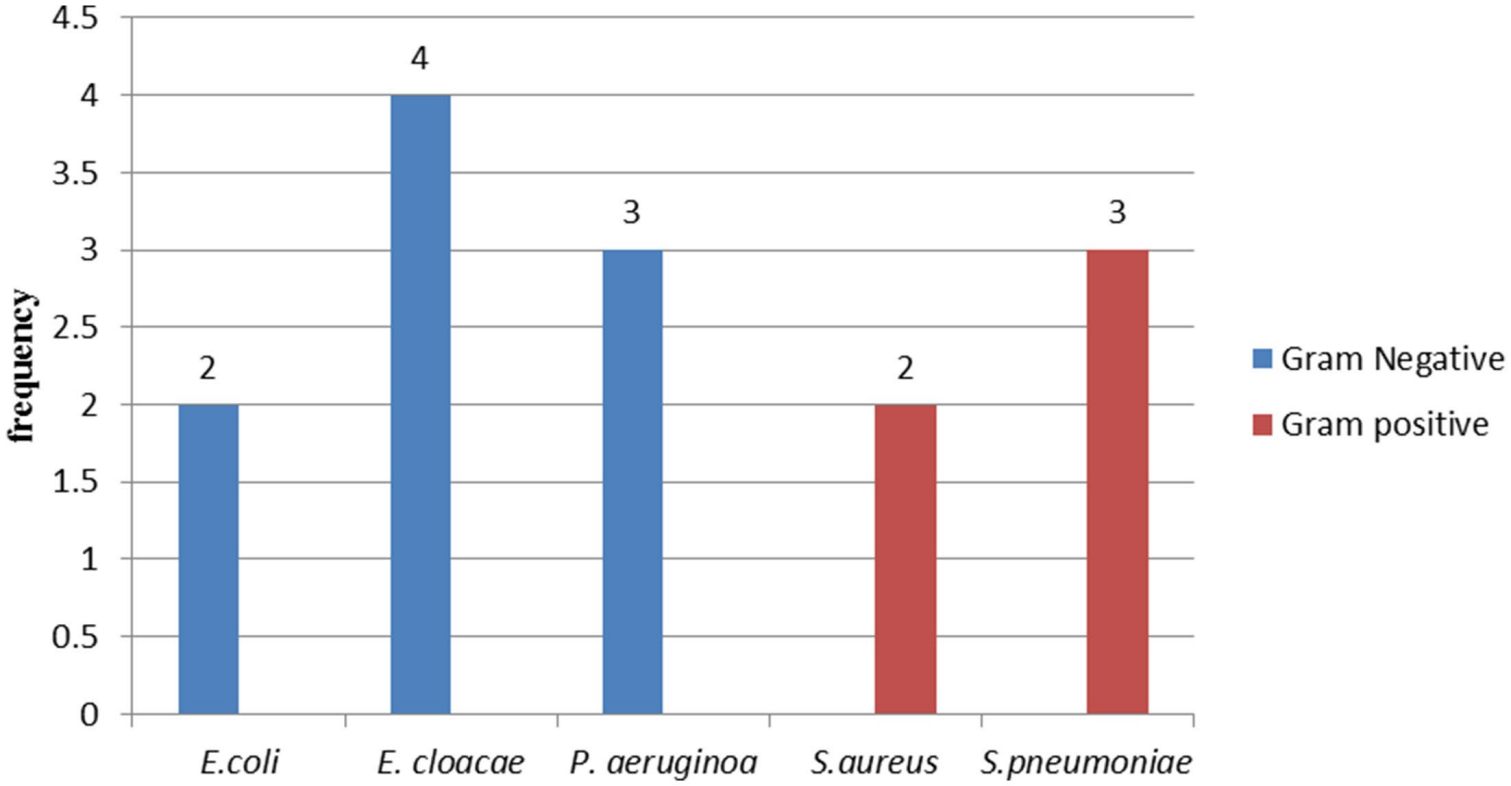
Bacterial isolates from patients diagnosed with sterile body site infections at DMCSH, from June 2022 to August 2022.

From a total of 187 specimens, 14 were culture-positive with overall prevalence of 7.5% (14/187) (95% CI: 3.72 to 11.27%). Of these 35.71% (5/14) were from age group below 20 years old. Most of the isolated bacteria were Gram-negative with 64.28% (9/14). Among these, *E. cloacae* 28.57% (4/14) and *P. aeruginosa* 21.42% (3/14) were the most predominant, while the remaining cases were Gram-positive cocci and account 37.72% (5/14). Of these, 21.42% (3/14), 14.28% (2/14) were *S. pneumoniae* and *S. aureus*, respectively (Figure 1). The most common bacterial isolates found in peritoneal fluid were *E. cloacae*, *S. aureus*, *S. pneumoniae*, and *E. coli*. Of these bacterial isolates, *E. cloacae* followed by *S. aureus* accounted for the highest percentage. *P. aeruginosa* was the most common bacterial isolated in CSF, followed by *E. coli* and *E. cloacae*. *S. pneumoniae* was the most common isolated from pleural fluid, followed by *P. aeruginosa*. No bacteria were isolated from synovial fluid.

### Antimicrobial susceptibility pattern for Gram-negative isolates

Most effective antimicrobials against *E. cloacae* isolates (*n* = 4) were amikacin (100%) and chloramphenicol (75%), imipenem (75%), and piperacillin/tazobactam (75%). In contrast, the highest resistance to cefuroxime, cefepime, and trimethoprim-sulfamethoxazole (75%) was observed against these isolates. The most effective antimicrobials against *P. aeruginosa isolates (n = 3)* were amikacin (100%), tobramycin (100%), and piperacillin/tazobactam (100%). Although, the highest resistance to gentamycin (66.7%) was observed against these isolates. All the *E. coli* isolates (*n* = 2) were susceptible to ceftazidime (100%), ceftriaxone (100%), tobramycin (100%), and chloramphenicol (100%). But they were 50%% resistant to cefuroxime, ciprofloxacillin, meropenem, imipenem, amikacin, and gentamycin (Table 2).

**Table 2:** Antimicrobial susceptibility patterns of Gram-negative isolates from patients suspected of sterile body site infections at DMCSH, from June 2022 to August 2022.

Bacterial isolate (n)	Pattern	AMP 10 μg	AMC 20/10 μg	CRO30μg	CXM 30 μg	FEP 30 μg	CAZ 30 μg	CPR 5 μg	SXT1.25/23.5 μg	MEM 10 μg	IMP 10 μg	AMK 30 μg	GN10 μg	TOB 10 μg	CHL 30 μg	TZB 100/10 μg	PIP 100 μg
*E. coli* (02)	%S	0	0	100	50	50	0	50	50	50	50	50	50	100	100	50	ND
	%I	0	0	0	0	50	0	0	50	0	0	0	0	0	0	50	ND
	%R	100	100	0	50	0	100	50	0	50	50	50	50	0	0	0	ND
*E. cloacae* (04)	%S	0	0	50	25	0	50	50	0	50	75	100	50	50	75	75	ND
	%I	0	25	25	0	25	0	0	25	25	0	0	0	0	25	25	ND
	%R	100	75	25	75	75	50	50	75	25	25	0	50	50	0	0	ND
*P. aeruginosa* (03)	S	ND	ND	ND	ND	33.3	0	66.7	ND	33.3	33.3	100	33.3	100	ND	100	67.3
	I	ND	ND	ND	ND	0	0	0	ND	0	0	0	0	0	ND	0	33.3
	R	ND	ND	ND	ND	66.7	100	33.3	ND	66.7	66.7	0	66.7	0	ND	0	0
Total (06)	S	0	0	4	3	1	2	4	1	4	5	6	4	5	5	5	1
	I	0	1	1	0	2	0	0	2	1	0	0	0	0	1	2	1
	R	6	5	1	3	3	4	4	3	3	3	1	4	2	0	0	0

AMP, ampicillin; AMC, amoxicillin/clavulanic acid; CRO, ceftriaxone; CXM, cefuroxime; FEP, cefepime; CAZ, ceftazidime; CPR, ciprofloxacillin; SXT, trimethoprim-sulfamethoxazole; MEM, meropenem; IMP, imipenem; AMK, amikacin; GN, gentamycin; TOB, tobramycin; CHL, cholaramphincol; PIP, piperacillin; TZB, piperacillin-tazobactam; ND, not done.

#### Antimicrobial susceptibility pattern for Gram-positive isolates

*S. aureus* (*n* = 2) isolates were highly susceptible to gentamycin, ciprofloxacin, and clindamycin (100%). But, all *S. aureus* isolates were 100% resistant to cefoxitin (methicillin resistance *staphylococcus aureus*), doxycycline, and erythromycins. *S. pneumoniae* (*n* = 3) was 100% susceptible to clindamycin and chloramphenicol. On contrary, all *S. pneumoniae* 100% resistant to trimethoprim-sulfamethoxazole (Table 3).

**Table 3:** Antimicrobial susceptibility patterns of Gram-positive bacterial isolate from patients suspected of sterile body site infections at DMCSH, from June 2022 to August 2022.

Isolates (n)	Pattern	P 10 unit	FOX 30 μg	CPR 5 μg	SXT 1.25/23.75 μg	VA 30 μg	GN 10 μg	CLD 2 μg	CHL30μg	DOX 30 μg	TCY 30 μg	ERY 15 μg	OXA 1 μg
*S. aureus*	%S	0	0	100	100	NA	100	100	100	0	50	0	–
	%I	0	0	0	0	NA	0	0	0	0	0	0	–
	%R	100	100	0	0	NA	0	0	0	100	50	100	–
*S. pneumoniae* (03)	%S	66.7	NA	–	0	66.7	NA	100	100	66.7	33.3	66.7	66.7
	%I	0	NA	–	0	0	NA	0	0	0	33.3	0	0
	%R	33.3	NA	–	100	33.3	NA	0	0	33.3	33.3	33.3	33.3

Penicillin, FOX, cefoxitin; CPR, ciprofloxacillin; SXT, trimethoprim-sulfamethoxazole; VA, vancomycin GN, gentamycin CLD, clindamycin; CHL, cholaramphincol; DOX, doxycycline; TCY, tetracycline; ERY, erythromycin OXA, oxacillin NA, not applicable.

### Multi-drug resistance of bacterial isolate from sterile body fluids

Out of 14 isolated pathogenic bacteria, 78.57% (11/14) (95% CI: 56.30–99.70%) of them were found to have multidrug resistance (MDR). Among these isolated bacteria, Gram-positive and Gram-negative had shown multiple drug resistance of 60% (3/5) and 88.89% (8/9) respectively. Among Gram-negative bacteria isolates, all (36.36%) *E. cloacae* had multi-drug resistance. Similarly, *S. aureus* had shown 100% (2/2) multi-drug resistance among Gram-positive bacteria isolates.

### Factors associated with bacterial isolates of sterile body site infections

Patients in the inpatient department were 10.267 times more likely to be infected than outpatients [AOR =10.267, 95% CI: (2.37, 44.50), *p* value =0.002]. Similarly, patients with comorbidity were 6.31 times more likely to develop bacterial infections than those without comorbidity [AOR = 6.31, 95% CI: (1.487–26.771), *p* value =0.012]. Patients who consumed alcohol regularly were 5.075 times more likely to develop bacterial infections [AOR = 5.075, 95%CI:(1.157–22.258)], *p* value =0.03 (Table 4).

**Table 4:** Bivariate and multivariate analysis of factors associated with patients suspected of sterile body site infections at DMCSH, from June 2022 to August 2022.

Variables	Category	Culture growth (*n*)		Bivariable analysis		Multivariable analysis	
		Yes	No	COR (CI)	*p*-value	AOR (CI)	*p*-value
Sex	Male	7	96	1	1		
	Female	7	77	1.246 (0.566–5.114)	0.334		
Age	≤20	5	63	1.349 (0.308–5.916)	0.691		
	41–60	3	33	1.545 (0.294–8.121)	0.607		
	≥61	3	26	1.962 (0.370–10.40)	0.429		
	21–40	3	51	1			
Residence	Rural	7	98	1			
	Urban	7	75	1.307 (0.439–3.886)	0.631		
Educational status	Educated	7	108	1			
	Uneducated	7	65	1.662 (0.588–4.951)	0.362		
Patient setting	Inpatient	10	48	6.51 (1.721–23.813)	0.006	10.267 (2.37–44.50)	0.002
	Outpatient	4	125	1		1	
Duration of illness	≤7 days	2	70	1			
	>7 days	12	103	4.078 (0.885–18.783)	0.071		
Alcohol consumption	Yes	11	82	4.069 (1.097–15.096)	0.036	5.075 (1.157–22.258)	0.031
	No	3	91	1			
History of hospitalization	Yes	10	58	4.957 (1.49–16.486)	0.009		
	No	4	115	1		1	
Invasive procedures & surgery	Yes	11	63	6.402 (1.721–23.813)	0.006	3.693 (0.869–15.693)	0.077
	No	3	110	1		1	
Comorbidity	Yes	11	57	7.462 (2.003–27.805)	0.003	6.31 (1.487–26.771)	0.012
	No	3	116	1		1	
Smoke cigarettes	Yes	6	64	1.277 (0.754–6.84)	0.145		
	No	8	109	1			

## Discussion

In the present study, the overall prevalence of bacterial pathogens was 7.5% (14/187) (95% CI: 3.72 to 11.27%). This finding is comparable with previous studies from India (10.81%) (31), and Nepal (10.7%) (32). In contrast, it is higher than reports from India 2.8% (33). But lower than other studies in Mekelle, Ethiopia (20.2%), Harar, Ethiopia (16.7%) (8), Tikur Anbesa, Ethiopia (14.1%) (6), India (14.78%) (34), and Brazil (21.3%) (35). These variations can be attributed to differences in sample processing, seasonal variation, and the practice of infection control.

In this study, the most frequently isolated microorganisms were Gram-negative bacteria (64.3%). A similar finding is reported from previous studies in EPHI, Ethiopia (74.6) (36), and India (67.8%) (37). In contrast, studies from Mekelle, Ethiopia (52.3%) (5), Turkey (61.7%) (38), and India (85.2%) (34) reported that Gram-positive bacteria were found to be the most common isolates. This difference could be due to different study areas, hospital-acquired infections, and different standard infection prevention control.

In this study, *E. cloacae* was the most frequent bacterial etiologies causing sterile bodies infection, in contrast. *E. coli* was the most prevalent isolated bacteria in a previous study conducted in EPHI, Ethiopia (36), Harar, Ethiopia (8), India (33), and Nepal (32). The detection of *E. cloacae* may be an indication of an opportunistic pathogen associated with hospital-acquired infections, due to poor infection prevention practices such as overcrowding, lack of standard facilities with controlled ventilation, and poor sterilization of all gowns and surgical equipment.

In our study, amikacin (88.9%), cholaramphincol (83.3%), piperacillin/tazobactam (77.8%), and imipenem (75%) were found to be the most effective against Gram-negative isolates. Whereas ampicillin (0%), cefepime (11.3%), and trimethoprim-sulfamethoxazole (16.7%) were the least effective antimicrobials against those Gram-negative isolates. This finding is comparable to previous studies in EPHI, Ethiopia (36), and India (31). This may be due to frequent use of these antimicrobials, the practice of self-medication, limited diagnostic facilities, and inappropriate antibiotic use.

In our study, all Gram-positive isolates were resistant to penicillin and cefoxitin, but most of the isolates were susceptible to erythromycins, doxycycline, and tetracycline. Similarly, high levels of resistance to beta-lactam agents were reported in previous studies in Ethiopia (8, 36) and India (39). This may be due to the presence of mobile-resistant factors, widely usage of these drugs in the treatment of different infections, and irrational use of antimicrobials.

All *S. aureus* isolates were methicillin-resistant (MRSA), similar to the study conducted in Harar, Ethiopia (8). But this finding is higher than study conducted in India, 36.6% (34), Nepal (30%) (32), and Addis Ababa, Ethiopia (50%) (6). This may be due to geographic variation, differences in infection control practice, and differences in treatments used. This may be related to various factors. As evidenced, for example, by this study, patients in the inpatient department had an infection rate that was 10.267 times higher than that of outpatients [AOR =10.267, 95% CI: (2.37, 44.50), *p* value =0.002]. Similarly, individuals with co-morbidity had a 6.31-fold increased risk of bacterial infections [AOR = 6.31, 95% CI: (1.487–26.771), *p* value =0.012] compared to those without co-morbidity.

In this study, high rates of multi-drug resistance (MDR) (78.57%) (95% CI: 56.30–99.70%) were reported, which is comparable to the findings in Addis Ababa, Ethiopia (75%) (6, 36), Harar, Ethiopia (76.4%) (8), Mekelle, Ethiopia (90%) (5) and India (62.9%) (40). But higher than study conducted in Nepal (40%) (32). These variations may be due to different bacterial isolates, patients’ awareness of the use of the antimicrobials, the difference in antibiotic prescribing policies, easy availability of some drugs without a prescription, isolated strain difference, and indiscriminate use of common antimicrobials. In addition, poor infection control practice (the use of alcohol, bleach, disposable gloves, sanitizer), along with poor hospital sanitation conditions, waste management guidelines, and inadequate infrastructure, raise serious concerns in Ethiopia since they may contribute to the emergence of drug resistance.

Being an inpatient, comorbidity, and the habit of drinking alcohol had a statistically significant association with bacterial infection of sterile body fluids. This finding is consistent with a study from Harar, Ethiopia (8). This could be due to the hospital’s level of environmental hygiene and surgical invasive procedures. Another significant factor associated with bacterial infection of sterile body fluids was having comorbidities. This might be due to low immune status due to any underlying medical conditions.

Drinking alcohol was also statistically significant to bacterial infection of sterile body fluids. This result agrees with studies in Arba Minch, Ethiopia (25). The reason might be due to common alcohol consumption influences the contribution of the intestinal microbial populations by way of disturbing the stability of intestinal homeostasis and impairs the phagocytic function of macrophages and neutrophils recruitment, as well as minimizes bacterial clearance inside the lungs.

In the present study, the main shortcomings were the limited sample size and inadequate hospital infrastructure. The microbiology laboratory, for example, lacks anaerobic culture, which is expensive and requires specialized equipment. Hence, the identification was limited to anaerobic bacteria because only aerobic cultures were carried out. This might be one of the reasons for the low culture-positive yield in this study.

### Conclusion

*E. cloacae*, *P. aeruginosa,* and *S. pneumoniae* were the most frequently isolated bacteria from various sterile bodily fluids. Antibiotics resistance was high among Gram-negative isolates. The majority of the isolates have shown multidrug resistance (MDR. Being an inpatient, comorbidity, and drinking alcohol were significant predictors for bacterial infection of sterile body fluids. Thus, there should be; regular monitoring of antimicrobial resistance patterns of infected body fluids, periodic antimicrobial surveillance, effective antibiotic policy with good medical practices, and proper infection prevention practices to control the disease transmission. Furthermore, as this study was done in a single study area, a multicenter study should be done to improve the clinical utility of the study.

## Data availability statement

The original contributions presented in the study are included in the article/supplementary material, further inquiries can be directed to the corresponding author.

## Ethics statement

The studies involving humans were approved by Ethical Review Committee of the College of Health Science, Debre Markos University. The studies were conducted in accordance with the local legislation and institutional requirements. The participants provided their written informed consent to participate in this study.

## Author contributions

DA: Conceptualization, Data curation, Formal analysis, Investigation, Methodology, Resources, Software, Writing – original draft, Writing – review & editing. GD: Conceptualization, Data curation, Formal analysis, Investigation, Methodology, Project administration, Software, Supervision, Validation, Visualization, Writing – review & editing. AA: Conceptualization, Formal analysis, Investigation, Methodology, Visualization, Writing – review & editing. AE: Conceptualization, Data curation, Formal analysis, Investigation, Methodology, Project administration, Resources, Software, Supervision, Validation, Visualization, Writing – review & editing.
